# Asiaticoside Combined With Carbon Ion Implantation to Improve the Biocompatibility of Silicone Rubber and to Reduce the Risk of Capsule Contracture

**DOI:** 10.3389/fbioe.2022.810244

**Published:** 2022-05-12

**Authors:** Xing Liu, Ya-Jun Song, Xing Chen, Meng-Ya Huang, Chen-Xi Zhao, Xun Zhou, Xin Zhou

**Affiliations:** ^1^ Department of Cosmetology, Chongqing Hospital of Traditional Chinese Medicine, Chongqing, China; ^2^ Department of Urology, Xinqiao Hospital, The Army Medical University, Chongqing, China; ^3^ Department of Pathology, Bishan Hospital, The Chongqing Medical University, Chongqing, China

**Keywords:** asiaticoside, silicone rubber, capsule contracture, carbon ion implantation, TGFβ/SMAD signaling pathway

## Abstract

Capsular contracture caused by silicone rubber is a critical issue in plastic surgery that urgently needs to be solved. Studies have shown that carbon ion implant in silicone rubber (carbon silicone rubber, C-SR) can significantly improve the capsular structure, but the effect of this improvement only appear 2months or later. In this study, asiaticoside combined with carbon silicone rubber was used to explore the changes in the capsule to provide a reference for the treatment of capsule contracture. Human fibroblasts (HFF-1) were used for *in vitro* experiments. The combined effect of asiaticoside and carbon silicone rubber on cell proliferation was determined by the CCK8 method, cell migration changes were measured by Transwell assays, cell cycle changes were measured by flow cytometry, and the expression levels of fibroblast transformation markers (vimentin and α-SMA), collagen (Col-1A1) and TGF-β/Smad signaling pathway-related proteins (TGF-β1, TβRI, TβRII and Smad2/3) were detected by immunofluorescence. *In vivo* experiments were carried out by subcutaneous implantation of the material in SD rats, and asiaticoside was oral administered simultaneously. WB and ELISA were used to detect changes in the expression of TGF-β/Smad signaling pathway-related proteins. TGF-β/Smad signaling pathway proteins were then detected and confirmed by HE, Masson and immunohistochemical staining. The results shown that asiaticoside combined with carbon ion implantation inhibited the viability, proliferation and migration of fibroblasts on silicone rubber. *In vitro* immunofluorescence showed that the secretion levels of α-SMA and Col-1A1 were significantly decreased, the transformation of fibroblasts into myofibroblasts was weakened, and the TGF-β/Smad signaling pathway was inhibited. *In vivo* experimental results showed that asiaticoside combined with carbon silicone rubber inhibited TGF-β1 secretion and inhibited the TGF-β/Smad signaling pathway, reducing the thickness of the capsule and collagen deposition. These results imply that carbon silicone rubber combined with asiaticoside can regulate the viability, proliferation and migration of fibroblasts by inhibiting the TGF-β/Smad signaling pathway and reduce capsule thickness and collagen deposition, which greatly reduces the incidence of capsule contracture.

## 1 Introduction

Silicone rubber has been widely used in the medical field since its introduction in the 1970s because of its biological inertness, corrosion resistance, wear resistance, and long-term persistence in the body (Duteille et al., 2018). Silicone rubber has become the most commonly used clinical filling material for soft tissue defects, and it is effective for repairing chest or facial deformities caused by congenital malformation, trauma or cancer resection (Yoon and Chang, 2020). However, due to the surface characteristics of silicone rubber, the R-group is located outside the spiral structure, which leads to strong hydrophobicity and poor biocompatibility of the material surface (Wang et al., 2014; Zhou et al., 2016). After long-term implantation *in vivo*, the surrounding tissues easily form fiber capsules. The formation of a capsule easily leads to subsequent capsular contracture, resulting in the deformation, displacement and even exposure of implant materials (Mazzocchi et al., 2012; Vieira et al., 2016; Virden et al., 2020). Capsular contracture is the most common and serious complication after breast augmentation, with an incidence of 3.6%–22.8% (Steiert et al., 2013). Capsular contracture leads to prosthesis hardening, breast swelling, pain and deformity and seriously affects patient quality of life (Baker et al., 2020). At present, the clinical treatments available for capsular contracture are poor. Once capsular contracture occurs, the only available treatment is the removal of the prosthesis through secondary surgery, which causes physical and mental damage to patients.

To improve this situation, numerous technologies have been developed to modify silicone rubber (Kang et al., 2018; Shin et al., 2018; Lam et al., 2021). It has been reported that ion implantation technology can significantly improve the capsular structure after implantation and reduce the probability of capsular contracture (Wang et al., 2014; Zhou et al., 2016). However, the silicone rubber material after ion implantation is only changed on the material surface, which has an obvious initial effect on tissue, but the effect on the capsule is not sustained long-term.

The formation of a capsule around the implant material is the result of physiological wound healing after implantation (Williams, 2008). The body cannot eliminate foreign bodies through phagocytosis. Capsule formation is a complex protective physiological response to isolate foreign bodies. It is initially characterized by an inflammatory response. After inflammatory stimulation, fibroblasts proliferate and migrate to the implant material surface. Then, the cytoskeleton rearranges and adheres closely to the implant material surface. A large number of fibroblasts arranged in a disorderly manner, resulting in the formation and thickening of fiber capsules (Mempin et al., 2018). Over time, the number of inflammatory cells gradually decreases, and cells begin to secrete large quantities of extracellular matrix (mainly collagen). Additionally, the powerful contractile function of fibroblasts pulls on the capsule tissue, resulting in capsule contracture. Inhibiting the excessive proliferation of fibroblasts on the surface of silicone rubber is the key to solving the problem of capsule contracture (Yoo et al., 2018).

*Centella asiatica* (L.) Urb. can inhibit the release of basic fibroblast growth factor by cells and macrophages, inhibit the proliferation of fibroblasts, promote scar apoptosis, and reduce the number of immune cells and loosen collagen fibers (Bylka et al., 2014; Sun et al., 2020). Its asiaticoside extract has an obvious curative effect on wound healing and fibrosis in scleroderma patients (Bylka et al., 2013; Phaechamud et al., 2015). Asiaticoside is one of three triterpenoid saponins isolated from *Centella asiatica* (L.) Urb. It can effectively prevent the occurrence of hypertrophic scars and keloids by inhibiting the proliferation, synthesis and secretion of fibroblasts (Qi et al., 2008; Ju-Lin et al., 2009; Tang et al., 2011; Lee et al., 2012). Also, because of its good safety profile, it can remain in the body for long periods with minimal adverse reactions. At present, a variety of dosage forms have been developed and widely used in the clinic and are administered orally, externally, and by intravenous drips (Qi et al., 2008; Bylka et al., 2013; Bylka et al., 2014; Namviriyachote et al., 2020). Due to the similarity between hypertrophic scarring and capsule contracture, combined with the advantage that asiaticoside can be administered orally long-term, in this study, we modified silicone rubber by carbon ion implantation technology and simultaneously administered treatment with asiaticoside to observe the changes in the capsule and reveal the molecular regulatory mechanism of asiaticoside on the capsule. This study was conducted to provide a reference for the clinical application of asiaticoside for solving the problem of capsule formation and capsular contracture.

## 2 Materials and Methods

### 2.1 Cell Culture and Preparation of Materials

The preparation methods of the carbon silicone rubber materials were performed as previously described (Wang et al., 2014; Zhou et al., 2016). Briefly, the smooth silicone rubber raw materials (Shanghai Winner Plastic Surgery Products Co., Ltd., Shanghai, China) was took graphite as carbon ion source and injected carbon ions with 10 KeV energy by universal ion implanter (Chengdu Institute of Nuclear Industry Physics, Chengdu, China). After that, the silicone rubber raw materials (SR) and carbon silicone rubber (C-SR) were cut into small discs with the size of 6-well plate, 24 well plate and 96 well plate with a punch. After cleaning and disinfection, they were laid on the bottom of the well plate. The cells used in this study were human fibroblasts (human foreskin fibroblasts-1, HFF-1), which were purchased from Beijing Dingguo Biotechnology Co., Ltd. The cells were cultured in DMEM containing 10% FBS, 100 U/ml penicillin and 80 U/ml streptomycin. The fibroblasts were routinely cultured at 37°C. When fibroblasts grew to approximately 90% confluence, they were digested with 2.5 g/L trypsin and subcultured at a 1:3 ratio. Third-generation fibroblasts in the logarithmic growth stage were inoculated with 1 × 10^4^ cells/ml in a 60 mm Petri dish, cultured in DMEM containing 10% FBS, and subjected to subsequent experiments after the cells grew to 50%–60% confluence. In this study, asiaticoside (AS) was purchased from Chengdu Desite Biotechnology Company (Chengdu, China) and Human TGF-β1 Recombinant Protein (#75362) was purchased from Cell Signaling Technology, Inc. The working concentration of asiaticoside is 500 mg/L and TGF-β1 is 10 ng/ml, which is prepared with culture medium in advance, and then the cells are treated with the culture medium. Cell experiments were divided into the following six groups: 1) the SR group; 2) the C-SR group; 3) the SR + TGF-β1 group; 4) the C-SR + TGF-β1 group; 5) the SR + TGF-β1 + AS group; and 6) the C-SR + TGF-β1 + AS group.

### 2.2 CCK8 Assays

Logarithmic growth phase fibroblasts were plated in a 96-well plate at a density of 1 × 10^4^ cells/well. When the cells adhered to the wall with different materials and grew to 70% confluence, they were starved with serum-free medium for 24 h. After treatment with 500 mg/L asiaticoside or/and 10 ng/ml TGF-β1 for 72 h, 20 μl of CCK-8 reagent was added to each well. After incubation for 4 h, the absorbance of the cells at a wavelength of 450 nm was measured with a microplate reader.

### 2.3 Cell Migration Experiment

Fibroblasts were cultured on each material for 24 h and treatment with 500 mg/L asiaticoside or/and 10 ng/ml TGF-β1 for 72 h to prepare a 1 × 10^5^ cells/ml serum-free cell suspension, plated in the upper chamber of a Transwell plate with an 8 μm pore size, and 600 μl serum-free medium was added to the lower chamber. Then, 500 mg/L asiaticoside was added to the lower chamber according to the group. After culturing for 24 h, the cells were removed, fixed with paraformaldehyde, and stained with crystal violet, and images of five fields were randomly taken from each well under a microscope to count the number of migrating cells.

### 2.4 Cell Cycle Detection

Fibroblasts at 1 × 10^5^ cells/well were cultured in a 6-well plate prepared with silicone rubber or carbon silicone rubber. After the corresponding treatment with 500 mg/L asiaticoside or/and 10 ng/ml TGF-β1 for 72 h, the cell density reached 80%–90% confluence after 24 h of culture. The cells were digested by trypsin, collected and centrifuged at 1,000 r/min for 5 min. The cells were washed twice with 1 ml of precooled PBS, transferred to a 15 ml centrifuge tube, added dropwise to 3 ml of precooled 70% ethanol, and fixed overnight at −20°C. The cells were then centrifuged at 800 r/min after fixation and washed 3 times for 5 min to remove ethanol, and 10 μl DNase free RNase was added. The cells were then incubated at 37°C for 30 min, centrifuged at 1,500 r/min for 5 min. The supernatant was discarded and 500 μl PI staining solution was added to each tube. The cells were then incubated at 37°C for 1 h, and the cell cycle distribution was detected by flow cytometry. The experiment was repeated three times, and the experimental data were analyzed.

### 2.5 Immunofluorescence

Logarithmic growth phase fibroblasts were inoculated into a Millicell chamber with different materials at a density of 1 × 10^4^ cells per well. When the cells grew to 70% confluence, they were starved with serum-free medium for 24 h. After 72 h of treatment with 500 mg/L asiaticoside or/and 10 ng/ml TGF-β1 for 72 h, the cells were washed with PBS three times, fixed with paraformaldehyde for 30 min, and permeabilized with Triton X-100 for 20 min. The cells were then blocked with 5% bovine serum albumin (BSA) at room temperature for 1 h, and then incubated in primary antibody overnight at 4°C. The cells were washed three times with PBS and fluorescent secondary antibody (1:100, Invitrogen, United States) was added, followed by incubation at room temperature for 1 h. DAPI was applied to stain the nuclei after washing with PBS and the cells were subsequently photographed under the microscope. The primary antibodies were as follows: vimentin (1:50, Proteintech, China), α-SMA (1:20, Proteintech, China), Col-1A1 (1; 10, Novus, United States), TGF-β1 (1:200, Affinity, United States), TβRI (1:50, Abcam, United States), TβRII (1:50, Abcam, United States), and Smad2/3 (1:100, Affinity, United States).

### 2.6 *In Vivo* Experiments

#### 2.6.1 Animals and Treatment

The animal experiments performed for this study were approved by the Animal Ethics Committee of the Chongqing Hospital of Traditional Chinese Medicine. SD rats were divided into five groups of three rats (15 rats total) as follows: the sham operation group (no material implanted), the normal SR group, the C-SR, group, the SR + AS group, and the C-SR + AS group. The two kinds of silicone rubber were cut into a disc shape with a diameter of approximately 8 mm and sterilized for later use. After the SD rats were anesthetized, a longitudinal incision of approximately 1 cm was made on the back, and the subcutaneous tissue was bluntly separated to form a “skin bag.” The appropriate material was implanted into the “skin bag” of each rat depending on the group. No material was implanted in the sham operation group. After the operation, the vital signs of the rats were observed, and their activities after anesthesia and awakening were observed. The rats were routinely monitored to observe the healing of the incision, redness, swelling, and ulceration. The sutures were removed 1 week later. The asiaticoside group was given oral asiaticoside every day after the operation. The dosage is based on the body weight of rats and the standard of 100 mg/kg each time, once a day. The control group was treated with the same volume of PBS. Forty-five days after the operation, the tissues and materials around the incision were removed from all rats for testing.

#### 2.6.2 HE and Masson Staining

The removed material and surrounding tissues were fixed in 4% paraformaldehyde and samples were then processed for paraffin embedding, sectioning and staining according to standard histological methods. The HE staining in this study was carried out according to the protocol supplied with the C0105 HE staining kit from Beyotime Biotechnology Company, Shanghai, China. Masson trichrome staining was performed using the MST-8003 Masson trichrome staining kit from Fujian Maixin Biotechnology Company, Fujian, China, according to the manufacturer’s directions. For quantification of implant capsules, consecutive tissue sections stained with H&E and Masson’s trichrome were compared in parallel and capsule thickness was measured at five places along the implant-tissue boundary for each implant (*n* = 5 per group per time point), for a total of five sections for both the upper as well as lower poles or sides of the implants.

#### 2.6.3 Immunohistochemistry

The removed tissue was embedded in paraffin, dewaxed in water, and incubated at room temperature with 3% H_2_O_2_ for 10 min to eliminate endogenous peroxidase activity. The sections were rinsed with distilled water and soaked in PBS for 5 min twice. Next, the sections were blocked with 10% normal goat serum (diluted in PBS) and incubated at room temperature for 10 min. The serum was removed without washing. The primary antibody was added and the sections were incubated at 4°C overnight. An appropriate concentration of biotin-labeled secondary antibody was added and the sections were incubated at 37°C for 30 min followed by washing with PBS. The sections were then stained and fully rinsed with tap water, counterstained, dehydrated, cleared and mounted. The primary antibodies were as follows: vimentin (1:2,000, Proteintech, China), α-SMA (1:1,500, Proteintech, China), Col-1A1 (1; 10, Novus, United States), and PCNA (1:50, Boster, China).

#### 2.6.4 WB

The tissue around the material was frozen and milled in liquid nitrogen, and the cell protein was extracted with RIPA lysis buffer. After electrophoretic separation, the proteins were transferred to PVDF membranes, and the membranes were blocked with 5% skimmed milk powder solution at room temperature for 1 h. Next, the membranes were incubated with primary antibody at 4°C overnight. The membranes were washed with TBST 3 times for 10, the secondary antibody (antibody dilution ratio of 1:8,000) was added, and the membranes were incubated for 1 h. The membranes were then washed with TBST 3 times for 10 min. Luminescent reaction solution was added, and the protein expression content was determined. The primary antibodies were as follows: Smad2/3 (1:500, Affinity, United States), vimentin (1:500, Proteintech, China), α-SMA (1:500, Proteintech, China), Col-1A1 (1:500, Novus, United States), TGF-β1 (1:500, Affinity, United States), TβRI (1:500, Abcam, United States), TβRII (1:500, Abcam, United States), Smad2/3 (1:500, Affinity, United States), p-Smad2/3 (1:500, Affinity, United States), and β-actin (1:1,000, Abcam, United States).

#### 2.6.5 ELISA

Tissue cell lysates from each group were collected and repeatedly frozen and thawed to lyse the cells. The lysate was then centrifuged at 3,000 r/min for 20 min to collect the supernatant. The concentration of TGF-β1 in the supernatant of fibroblasts in each group was measured according the ELISA kit manufacturer’s directions. The absorbance was measured at 450 nm with an enzyme labeling instrument. The concentration of TGF-β1 was normalized and calculated according to the linear calibration curve obtained from measuring the standard solution.

### 2.7 Statistical Analysis

Each experiment was repeated independently at least 3 times, and GraphPad Prism software was used for data analyses. The data are expressed as the mean ± standard deviation. The difference between groups was calculated by a *t*-test and one-way ANOVA. For all comparisons, *p* < 0.05 was regarded as statistically significant.

## 3 Results

### 3.1 Asiaticoside Combined With Carbon Ion Implantation Inhibits TGF-β1-Mediated Fibroblast Viability and Cell Cycle Changes on the Surface of Silicone Rubber

**FIGURE 1:**
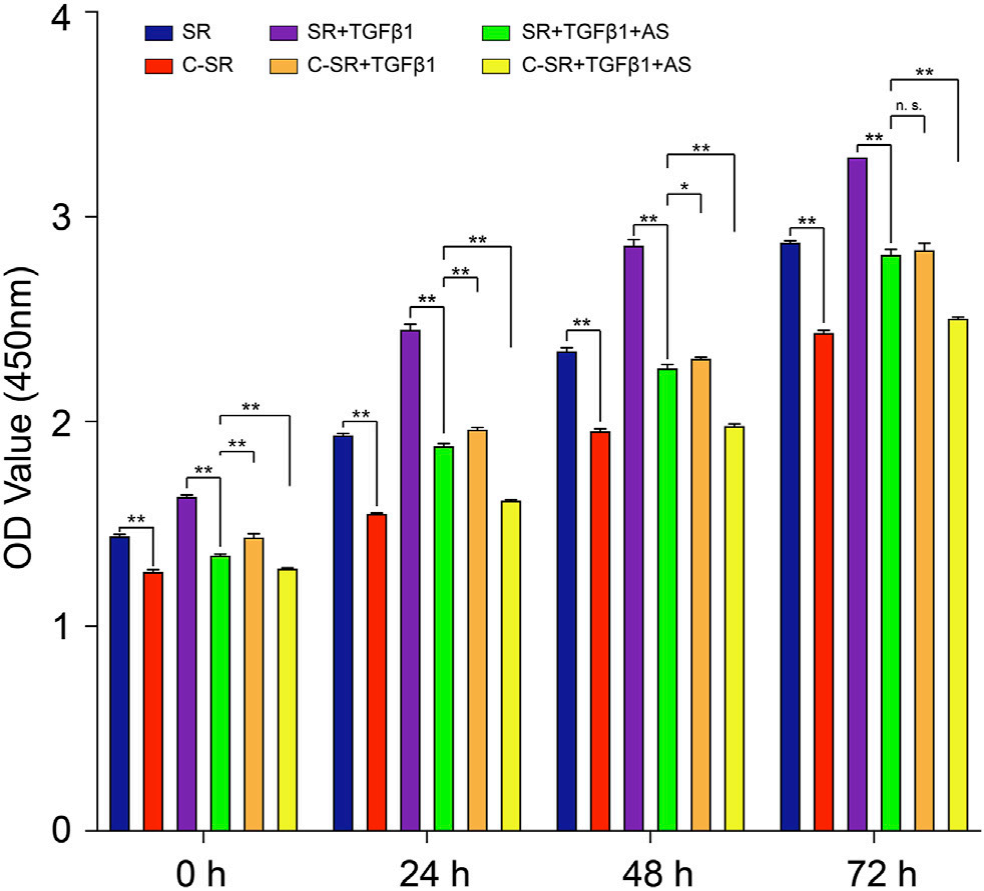
Asiaticoside combined with carbon ion implantation inhibits TGF-β1-mediated fibroblast viability. Cell viability of human fibroblasts on silicone rubber and carbon ion implantation silicone rubber determined by the CCK-8 assay. Absorbance was measured at 450 nm at 0, 24, 48, and 72 h. Significance was calculated by one-way ANOVA with Tukey’s *post hoc* test, ^∗^ *p* < 0.05, ^∗∗^ *p* < 0.01, and n. s. means no significant difference. (*n* = 5).

The CCK8 method was used to detect the changes in the viability of fibroblasts on the surface of the silicone rubbers, and the results are shown in Figure 1. Figure 1 shows that the viability of fibroblasts on the surface of carbon silicone rubber was lower than that of those on smooth silicone rubber. We further treated the fibroblasts with TGF-β1, which can simulate the development of fibroblasts toward fibrosis, and then observed changes in cell viability. The viability of fibroblasts on the surface of smooth silicone rubber and carbon silicone rubber both increased after TGF-β1 treatment, and these fibroblasts still demonstrated high activity after 72 h. After adding asiaticoside, the viability of fibroblasts on the surface of all materials showed a decreasing trend, and the difference was statistically significant. In addition, we found that the viability of fibroblasts on the surface of carbon silicone rubber with TGF-β1 treatment was even higher than that of those on the surface of smooth silicone rubber after TGF-β1 treatment and the addition of asiaticoside. However, by 72 h, there was no significant difference between the two groups. This finding further shows that asiaticoside can significantly reduce the viability of fibroblasts on the surface of silicone rubber.

**FIGURE 2:**
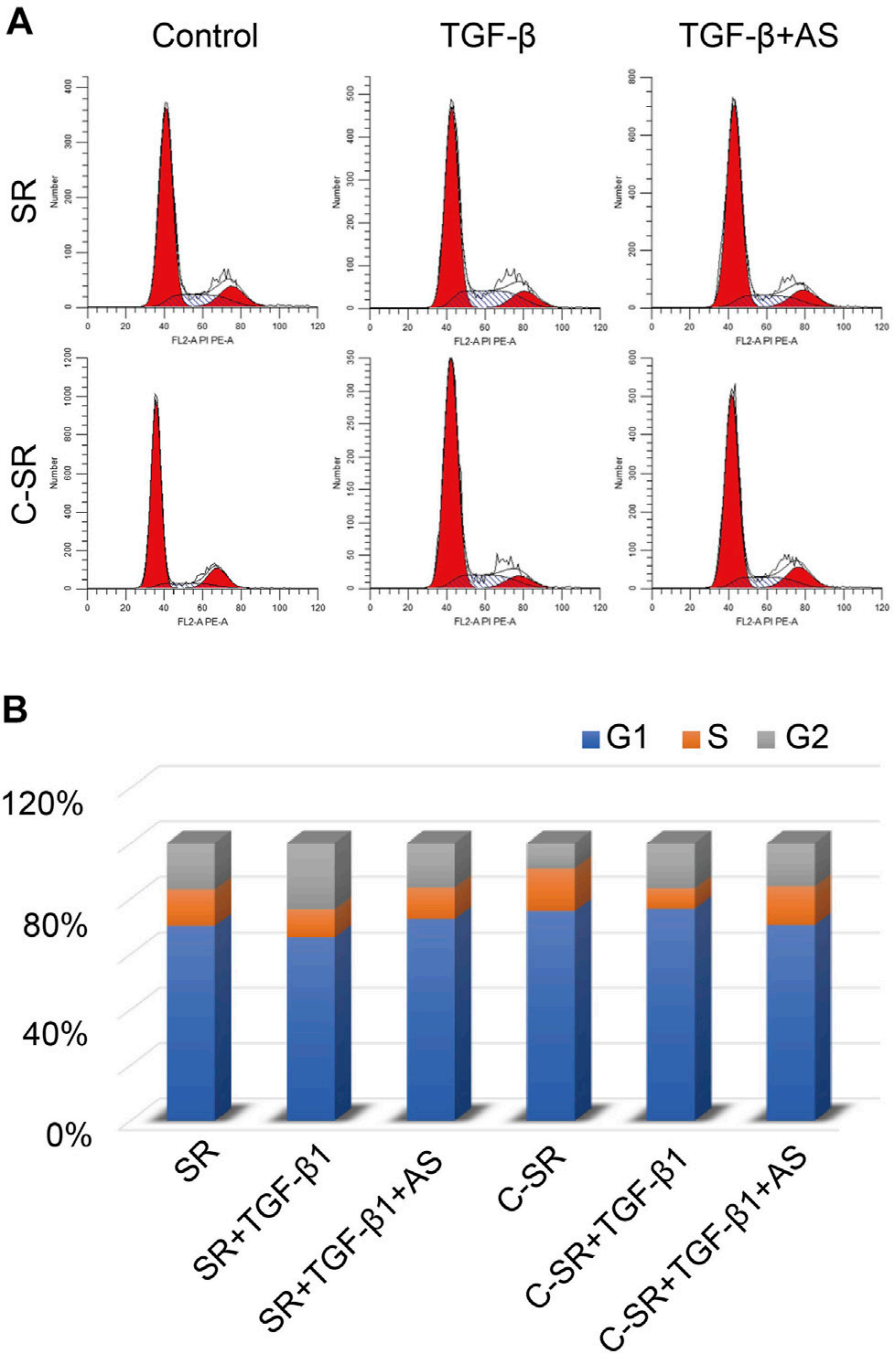
Asiaticoside induced G1 phase cell cycle arrest. **(A)** The peak plot shows the results of cell cycle analysis for human fibroblasts after treatment with asiaticoside. **(B)** The percentages of the human fibroblast population in G1, S, and G2/M phases after treatment with asiaticoside.

We further determined the cell cycle changes of the fibroblasts on the surface of the material before and after asiaticoside treatment by flow cytometry. The results are shown in Figure 2. The difference in the proportion of cells in the S phase of the cell cycle between the two materials was different. After TGF-β1 treatment, the difference in the number of S-phase fibroblasts between the two materials was still large, but this difference became significantly smaller after asiaticoside treatment. These results also show that asiaticoside plays an important role in inhibiting the proliferation of fibroblasts.

### 3.2 Asiaticoside Combined With Carbon Silicone Rubber Inhibits TGF-β1-Mediated Migration of Fibroblasts on the Surface of Silicone Rubber

**FIGURE 3:**
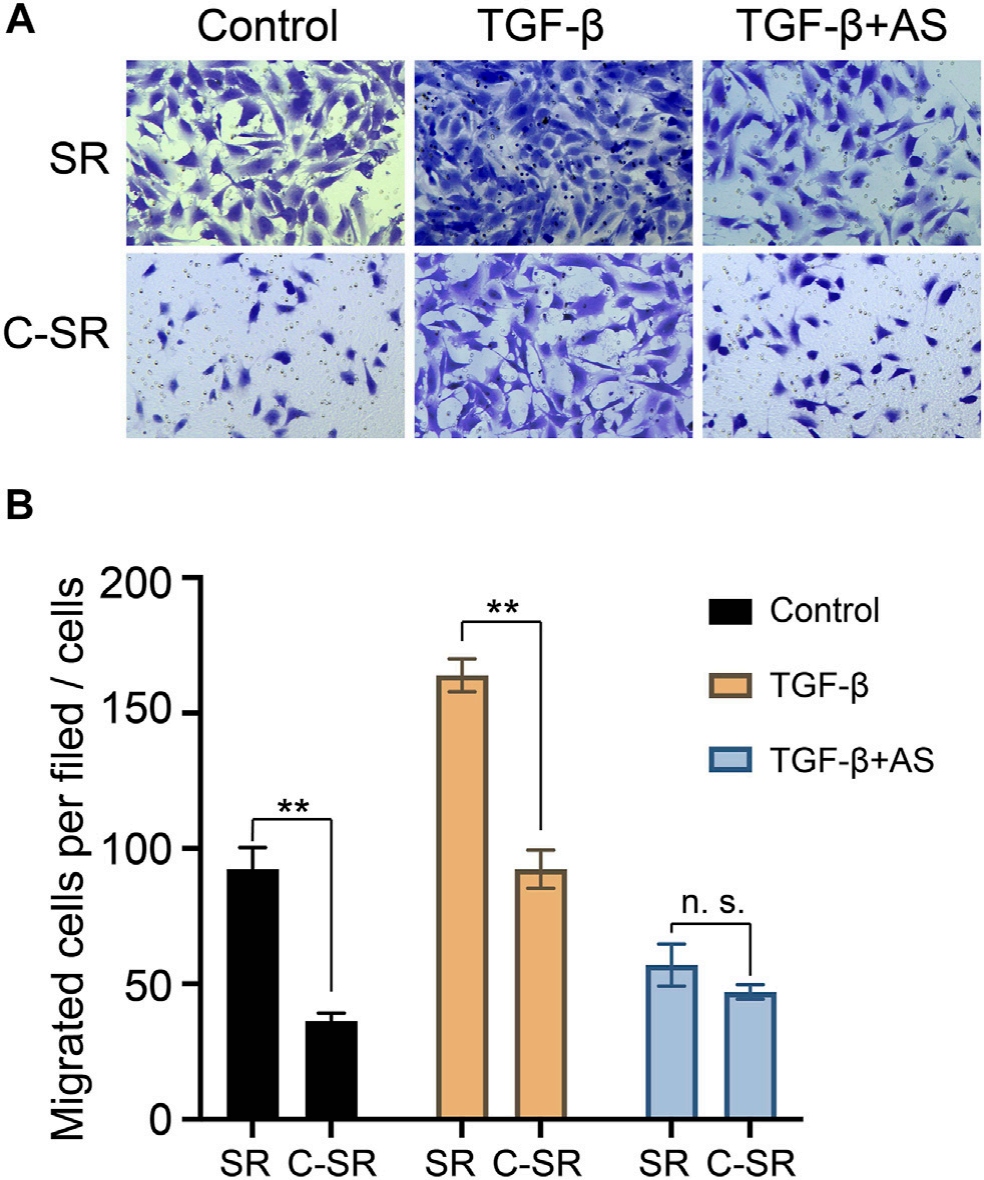
Effect of asiaticoside on TGF-β1-induced migration of human fibroblasts. Cell migration was evaluated using a Transwell chamber. The cells were pretreated with various doses of TGF-β1 for 60 min on silicone rubber (SR) and carbon ion implantation silicone rubber (C-SR) and then stimulated by asiaticoside. Photographs **(A)** and a histogram **(B)** showing the numbers of migrated cells in each panel are presented. Significance was calculated by *t*-test, ^∗∗^ *p* < 0.01, and n. s. no significant difference (*n* = 5).

Cell migration plays an important role in the early tissue repair process mediated by implanted materials. In this study, Transwell technology was used to explore the effect of asiaticoside on the migration ability of fibroblasts on the surface of each material. The results are shown in Figure 3. Fibroblasts had a significantly greater migration ability on silicone rubber than on carbon silicone rubber. After TGF-β1 treatment, the migration ability of fibroblasts on the surface of each material increased, but the cell migration ability on the surface of silicone rubber was still greater than that on carbon silicone rubber. Surprisingly, after the addition of asiaticoside, the difference in the migration ability of fibroblasts on the surface of silicone rubber and carbon silicone rubber disappeared. These results further indicate that asiaticoside can inhibit the migration of fibroblasts.

### 3.3 Asiaticoside Combined With Carbon Ion Implantation Regulates Myofibroblast Transformation and Collagen Secretion of Fibroblasts on Silicone Rubber Through the TGF-β/Smad Signaling Pathway

**FIGURE 4:**
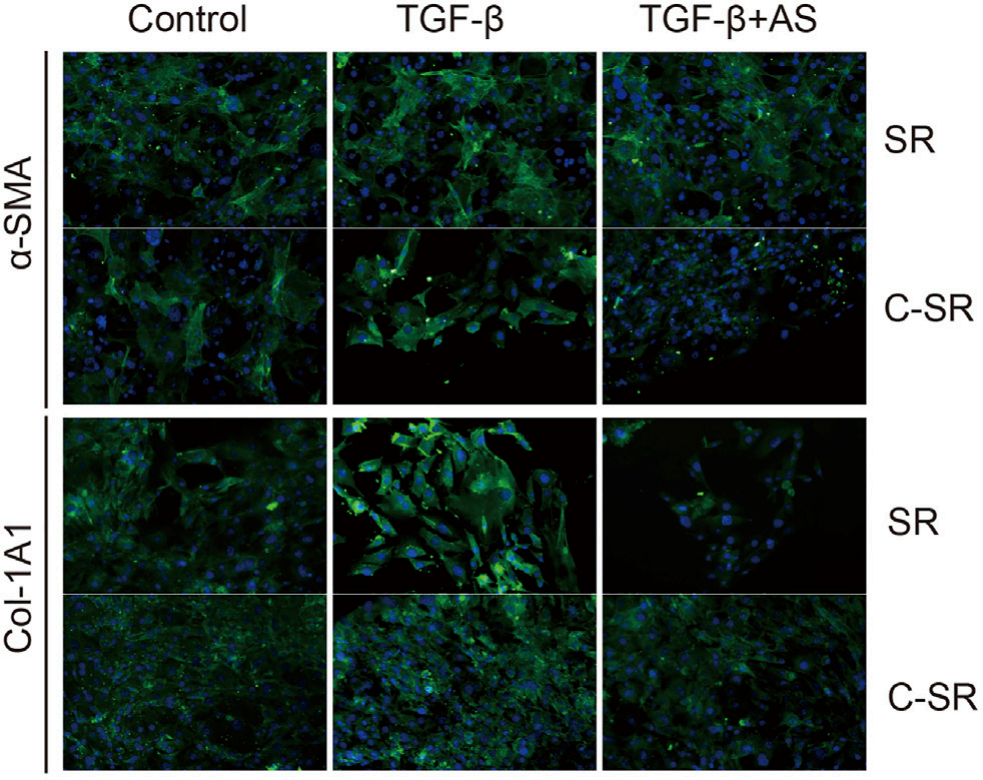
Effect of asiaticoside on TGF-β1-induced transformation of fibroblasts into myofibroblasts and collagen deposition. Immunofluorescence staining of human fibroblasts on silicone rubber (SR) and carbon silicone rubber (C-SR) with Col-1A1 and α-SMA (100X).

**FIGURE 5:**
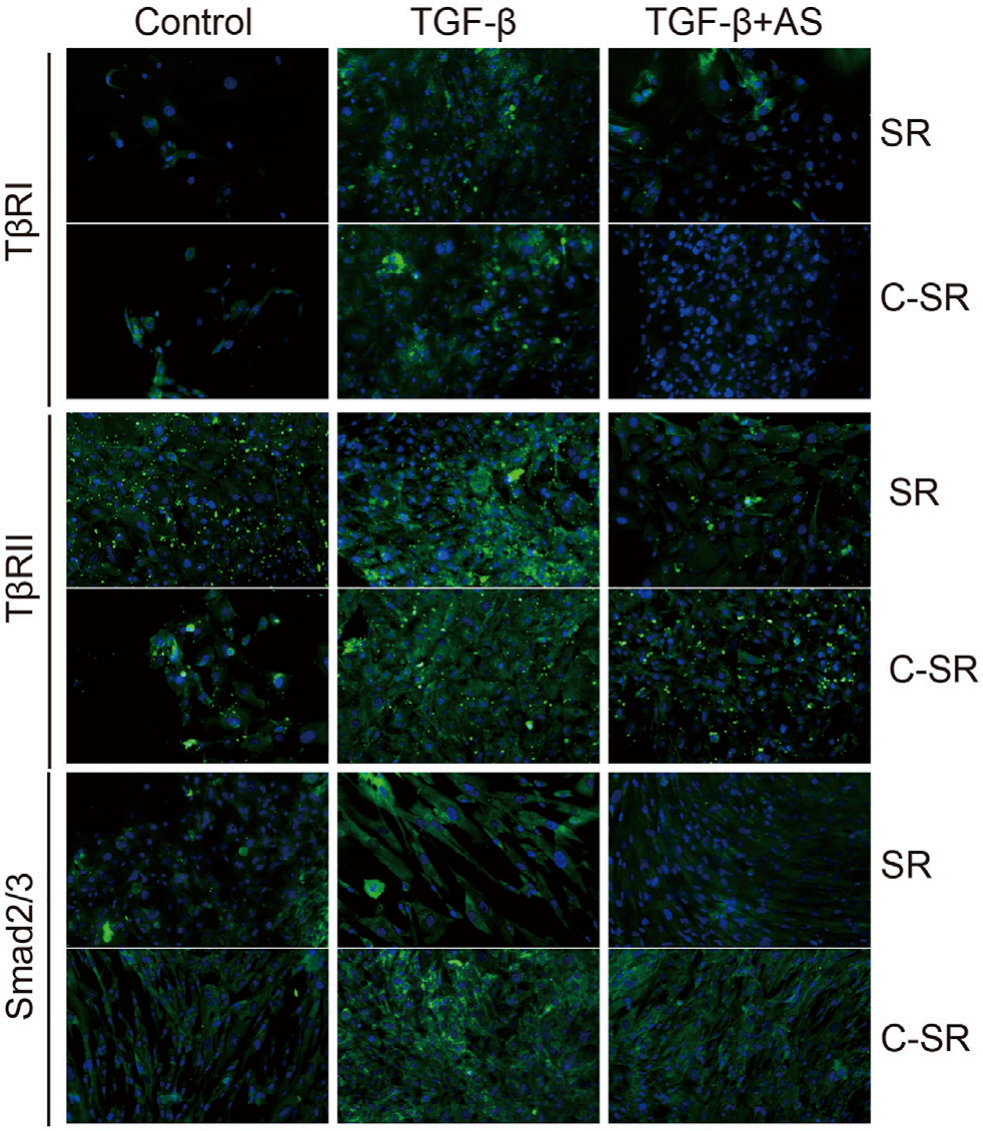
Effect of asiaticoside on TGF-β1-induced changes in TGF-β/Smad signaling pathway. Immunofluorescence staining of human epidermal fibroblasts on silicone rubber (SR) and carbon ion implantation silicone rubber (C-SR) with TβRI, TβRII and Smad2/3 (100X).

The transformation of fibroblasts into myofibroblasts is the key to the formation of the capsule around the implant material, and myofibroblasts are important source of collagen. Too many myofibroblasts leads to excessive collagen deposition, which further causes the capsule to thicken, harden and become prone to contracture. To observe the transformation of fibroblasts into myofibroblasts on the surface of various materials regulated by asiaticoside and explore the related signal regulation pathways, the expression changes in myofibroblast marker protein α-SMA and TGF-β/Smad signal pathway-related proteins were observed and analyzed by immunofluorescence. The expression levels of collagen were further compared. It can be seen from Figures 4,5 that after stimulation with TGF-β1, the expression of TGF-β/Smad signal pathway-related proteins TβRI, TβRII and Smad2/3 increased. In addition, we also observed that the secretion of collagen increased significantly after the addition of TGF-β1. However, when asiaticoside was added, the expression of each protein, especially TβRI, TβRII and Smad2/3, showed a decreasing trend. In addition, the secretion of α-SMA and collagen also decreased significantly, indicating that asiaticoside inhibits the myogenic transformation of fibroblasts and reduces the secretion of collagen through the TGF-β/Smad signaling pathway.

### 3.4 Asiaticoside Combined With Carbon Ion Implantation Technology Can Reduce Capsular Thickness and Collagen Formation Around Silicone Rubber Through the TGF-β/Smad Signaling Pathway *In Vivo*

**FIGURE 6:**
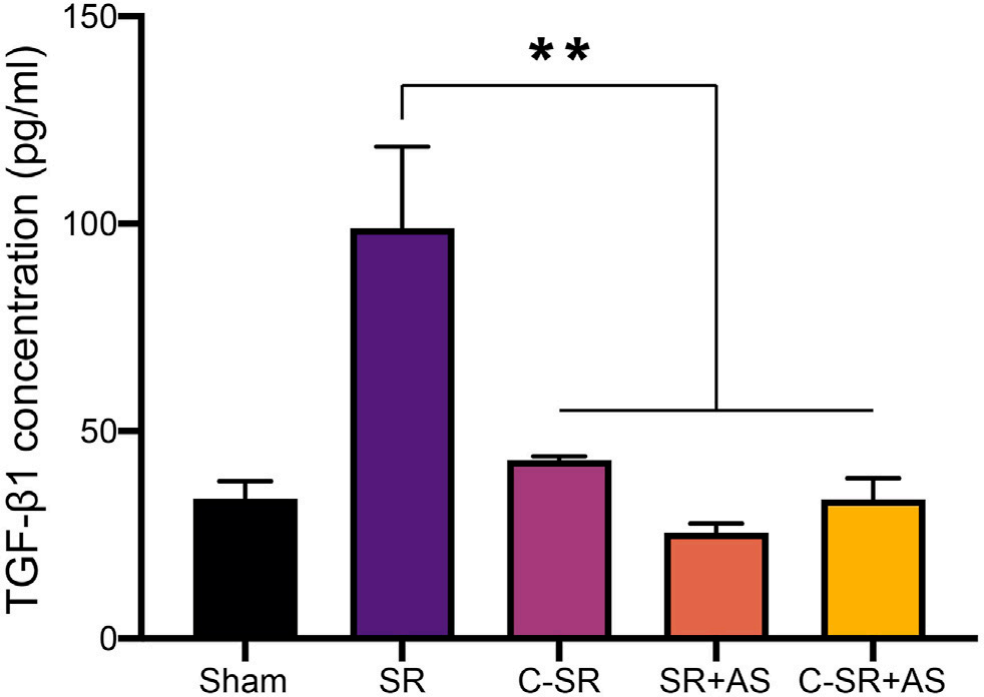
Effect of asiaticoside on secretion of TGF-β1 in tissue of silicone rubber (SR) and carbon silicone rubber (C-SR) implantation site in SD rats. Significance was calculated by one-way ANOVA with Tukey’s *post hoc* test, ^∗∗^ *p* < 0.01, (*n* = 5).

**FIGURE 7:**
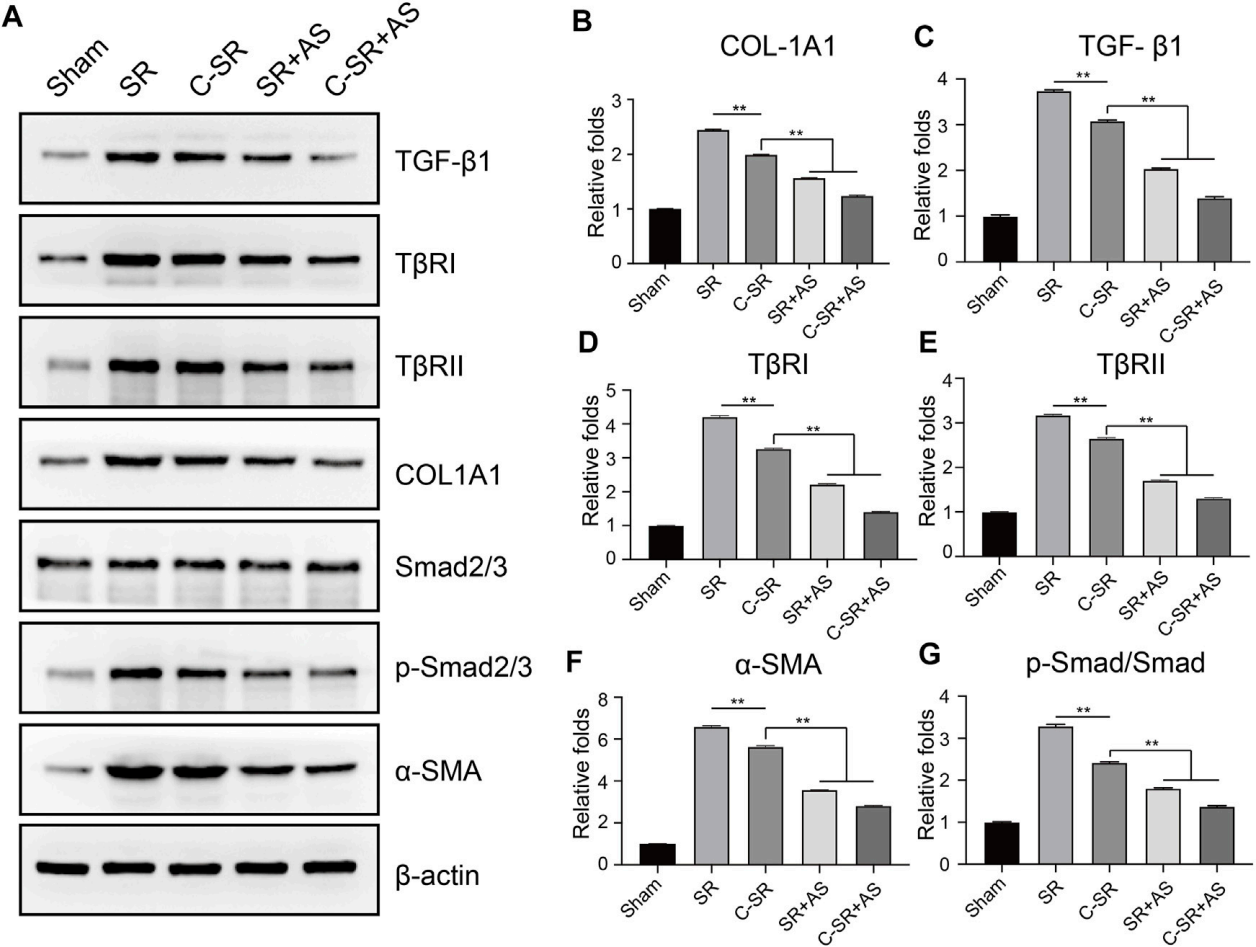
Effect of asiaticoside on transformation of fibroblasts into myofibroblasts, collagen deposition and changes in the TGF-β/Smad signaling pathway *in vivo*. **(A)** Representative Western blot analysis of TGF-β1, TβRI, TβRII, Col-1A1, Smad2/3, p-Smad2/3 and α-SMA. One representative blot of three experiments is presented. β-actin was used as loading control. **(B–G)** Protein signals in A were determined by densitometric analysis of Western blotting using ImageJ software (NIH). Data are reported as the mean ± standard error (*n* = 3, ^∗^ *p* < 0.05).

**FIGURE 8:**
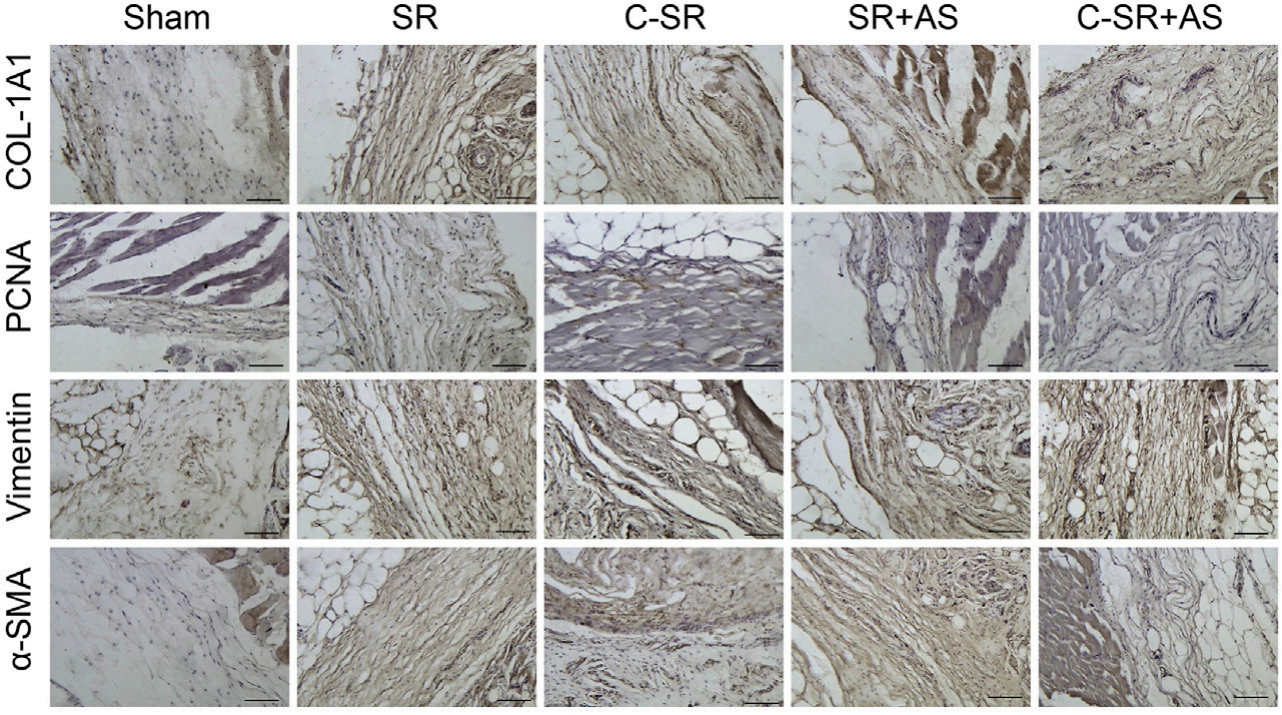
Effect of asiaticoside on the transformation of fibroblasts into myofibroblasts, collagen deposition and fibroblast proliferation *in vivo*. Immunohistochemical staining of tissue around silicone rubber (SR) and carbon silicone rubber (C-SR) with α-SMA, vimentin, PCNA and COL-1A1 (100X).

**FIGURE 9:**
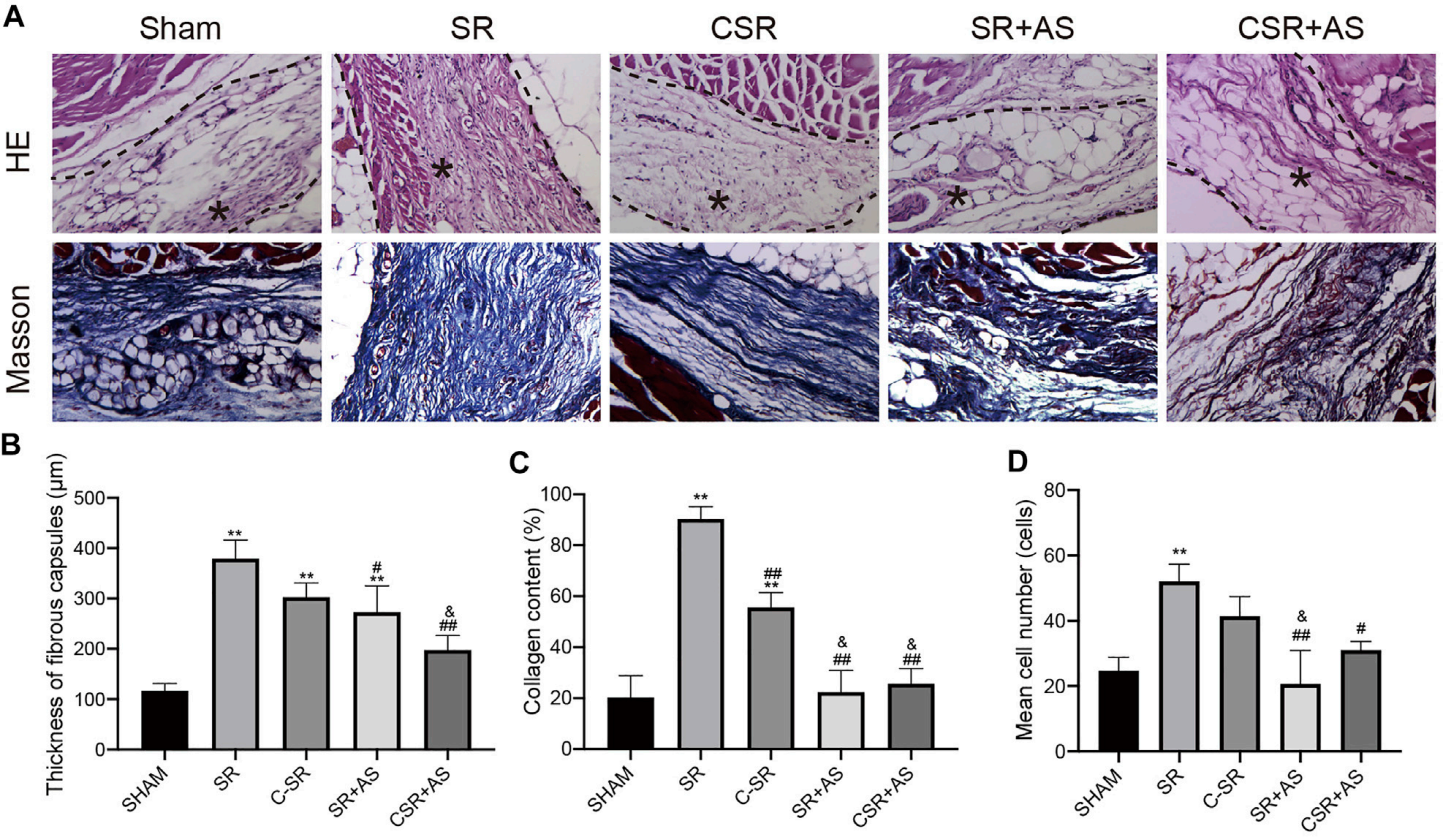
Capsular formation and collagen deposition. **(A)** Representative HE and Masson staining images of capsules around SR and C−SRs after 45 days in rats (400×). ∗ capsular tissue. **(B)** Capsule thickness. **(C)** Collagen content. **(D)** Quantification of macrophages. The data are presented as the mean ± SD, *n* = 5. One-way ANOVA and Tukey’s multiple comparisons tests were used, ^∗∗^ *p* < 0.01 vs. SHAM. ##*p* < 0.01, #*p* < 0.05 vs. SR. &*p* < 0.05 vs. C-SR.

To further confirm that asiaticoside inhibits the TGF-β/Smad signaling pathway, the effects of asiaticoside on TGF-β/Smad signaling pathway-related proteins, capsular thickness and collagen around the material were observed and analyzed *in vivo*, and the secretion changes in TGF-β1 were analyzed by ELISA. Figure 6 shows that the concentration and content of TGF-β1 secreted into the tissues surrounding carbon silicone rubber were significantly smaller than those secreted into the tissues surrounding silicone rubber. Furthermore, compared with those in the asiaticoside drug treatment group, the TGF-β1 levels were further reduced. These results indicate that asiaticoside can reduce the secretion of TGF-β1 by tissues. Furthermore, we also observed and analyzed the expression changes in other related proteins in the tissues around the materials by WB. Figure 7 shows that in the tissues surrounding carbon silicone rubber, the expression levels of TGF-β1, TβRI, TβRII, COL1A1, and α-SMA were lower than those in the tissues surrounding silicone rubber. In addition, we observed that the phosphorylation level of Smad2/3 showed a decreasing trend. Further tissue immunohistochemistry showed that the level of PCNA protein, which reflects cell proliferation, was lower in both the carbon silicone rubber group and the asiaticoside treatment group. The expression of collagen-related protein and α-SMA was also the lowest in the carbon silicone rubber combined with asiaticoside group. Vimetin protein is a marker protein of fibroblasts, in the asiaticoside treatment group, we observed a large number of fibroblasts aggregated (Figure 8). Combined with the expression of α-SMA protein, we can conclude that asiaticoside inhibited the formation of fibroblasts transformation, thereby inhibiting capsular contracture. The capsular structure of the tissue around the material was analyzed, and the result is shown in Figure 9. Figure 9 shows that the carbon silicone rubber has a thinner capsular structure than silicone rubber, and the asiaticoside treatment group is also thinner compared with the silicone rubber group. The treated groups all showed a thinner capsular structure, and the thickness was less than that of the carbon silicone rubber group. The capsule tissue of the asiaticoside treatment group was looser. In addition, the carbon silicone rubber group had less collagen deposition than that observed in the silicone rubber group. Collagen deposition was further reduced when carbon silicone rubber was combined with asiaticoside treatment. There were also significant differences in the quantitative results. Interestingly, we also observed a reduction in the number of inflammatory cells. These results also further reflect that asiaticoside combined with carbon ion implantation technology inhibits the proliferation of fibroblasts and myofibroblast transformation on the surface of silicone rubber through the TGF-β1/Smad signaling pathway, reduces the formation of capsular tissue, and further reduces collagen secretion. Thus, applying this therapeutic combination could play a substantial role in reducing the incidence of capsular contracture.

## 4 Discussion

Statistically, breast augmentation has ranked first among the top five cosmetic surgeries performed in the United States for 11 consecutive years, with 270,000 to 290,000 surgeries performed each year (Namviriyachote et al., 2020). However, approximately 25,000 cases of inner implantation material removal occur each year for various reasons (Xue and Pu, 2021), accounting for 8.78% of breast implantations (Headon et al., 2015; Coombs et al., 2019). Therefore, the prevention and treatment of breast implantation material complications has become an urgent problem to be solved in the field of cosmetic surgery. The most common complication, breast prosthesis capsule contracture, is of substantial concern. The U.S. Food and Drug Administration (FDA) reported that the incidence of capsular contracture after the first breast prosthesis implantation was 2%–15%, and the incidence of capsular contracture after the second breast prosthesis surgery increased to 5%–22% (Bengtson, 2021). Capsular contracture is the most common complication after breast augmentation involving prostheses (Wan and Rohrich, 2016), and is divided into four levels according to the Baker classification system. In grade I capsular contracture, the breast is naturally soft; in grade II, the tissue appears natural and slightly hard to the touch; in grade III, the tissue is deformed and hard to the touch, and in grade IV, the tissue appears severely deformed and is hard and painful. Grade III and IV capsular contracture seriously affect the outcome of breast augmentation surgery, and active treatment measures are required. This greatly increases the suffering on patients.

Many studies have revealed that the surface structure of silicone rubber has a certain influence on the occurrence of capsular contracture. The textured (rough) structure of the surface of silicone rubber is believed to destroy the contractile force of the tissues surrounding the silicone rubber (Adams, 2009; Stevens et al., 2013). Many meta-analyses also have confirmed that the risk of capsular contracture with the use of rough silicone rubber is significantly lower than with the use of smooth silicone rubber (Hwang et al., 2010). The coated collagen fibers of smooth surface silicone rubber are arranged in an orderly manner, whereas the coated collagen fibers of rough surface silicone rubber are disorderly, and the collagen density decreased with time with rough surface silicone rubber but increased with smooth surface silicone rubber (Wan and Rohrich, 2016). Some studies have also indicated that nano or micromorphology of the surface of silicone rubber formed by ion implantation technology can promote cell adhesion and reduce collagen deposition and coating thickness (Wang et al., 2014; Zhou et al., 2016). In this study, we used carbon ion-implanted silicone rubber combined with asiaticoside to significantly reduce the transformation of fibroblasts into myofibroblasts around the material, inhibit the excessive proliferation and migration of fibroblasts, and further reduce the formation of capsules and the deposition of collagen, which greatly reduced the incidence of capsule contracture.

*Centella aslatica* (L.) Urb, is a Chinese herbal medicine that yields minimal adverse reactions and is increasingly used in modern medicine. Histopathological examination previously revealed that asiaticoside can significantly inhibit dermal fiber proliferation and angiogenesis and inhibit the synthesis of collagen fibers, thus inhibiting scar (Bylka et al., 2014). Other studies have revealed that asiaticoside can reduce the expression of the TGF-β receptor in keloid fibroblasts and increase the expression of Smad7 in a dose-dependent manner to inhibit the production of ECM protein in the extracellular matrix (Tang et al., 2011; Liu et al., 2015). The mechanisms of keloid formation are very similar to those of capsule formation. In this study, we found that asiaticoside combined with carbon ion implantation into silicone rubber decreased TGF-β levels, inhibited the expression of the TGF-β receptor and further reduced collagen deposition. Interestingly, we found that asiaticoside combined with carbon ion implantation into silicone rubber can reduce collagen formation by reducing Smad2/3 phosphorylation.

TGF-β1 is the most important cytokine in the TGF family and is involved in the regulation of changes in capsule tissue. TGF regulates cell adhesion, granulation tissue degradation, and extracellular matrix synthesis and deposition by stimulating the differentiation, transformation and proliferation of fibroblasts (Lee et al., 2006; Munger and Sheppard, 2011; Juhl et al., 2020). TGF-β1, through paracrine and autocrine mechanisms, directly or indirectly acts on wound-repairing cells, resulting in important biological effects, including chemotaxis, migration, proliferation and differentiation, synthesis and secretion of extracellular matrix (Meyer-ter-Vehn et al., 2011). If the anti-inflammatory and proinflammatory activities of TGF-β1 are unbalanced, the capsule tissue will proliferate excessively and show excessive fibrosis (Schiller et al., 2004). Under pathological conditions, the function of myofibroblasts changes, producing excessive collagen fiber deposition. Myofibroblasts persist and display abnormal functions, which eventually leads to the formation of a thicker capsule and capsule contracture (Meng et al., 2016; Hu et al., 2018).

The main mechanism of TGF-β receptor signal transduction follows receptor-mediated phosphorylation and interaction. TGF-β first binds to the homodimer TGFβRII, which is a high-affinity receptor. This interaction causes a conformational change between the ligand and TGFβRII, thereby forming a new high-affinity binding site for TGFβRI. After recruiting two units of TGFβRI, type II receptor kinase phosphorylates the glycine and serine-rich serine residues in the proximal membrane subdomain of TGFβRI, thereby activating type I receptor kinase (Katzel et al., 2011; Shi et al., 2011). In this study, we also found when carbon ion silicone rubber was combined with asiaticoside, the expression levels of TGFβRI and TGFβRII were low, and the phosphorylation levels of SMAD2 and SMAD3 were reduced. Signal transmission from the TGF-β1 receptor to the nucleus mainly occurs through the phosphorylation of SMAD proteins in the cytoplasm (Yan et al., 2009). The TGF-β1 receptor can specifically recognize and phosphorylate SMADs. Activated SMAD2, SMAD3 and SMAD4 can cause capsule contracture and the fibrosis of capsule tissue *in vitro* and *in vivo*.

Approximately 45 days after implanting the material, fibroblasts will generate traction on the newly deposited collagen fibers, thereby gradually hardening the ECM (Derynck and Zhang, 2003). When the mechanical resistance of the ECM exceeds the force generated by such low-contraction early wounds, the level of TGF-β1 activity increases (Tomasek et al., 2002). Active TGF-β1 promotes the activation of fibroblasts into highly contracted myofibroblasts, promotes vascular maturation and inhibits the growth and migration of epithelial cells (Schiller et al., 2004). A series of changes are induced during the transformation of fibroblasts into myofibroblasts. TGF-β1 promotes the new expression of α-SMA subtypes, giving fibroblasts the required traction during the process of capsular contracture (Brunner and Blakytny, 2004; Sanders et al., 2015). In normal repair, these activities are terminated in part because the tissue’s TGF-β1 supply is depleted. Under the control of the complex internal environment, the myofibroblast population undergoes large-scale apoptosis (Khouw et al., 1999). If the apoptosis clearance mechanism fails, fibroblasts and inflammatory cells will continue to exceed the levels required for normal healing, resulting in an imbalance between normal repair and excessive repair, leading to tissue fibrosis and capsule hardening or cause capsule contracture (Hinz and Lagares, 2020). In an unstable internal environment, TGF-β1 no longer stimulates physiological healing but effectively promotes the occurrence of capsule contracture. Animal studies have revealed that a complete capsule can form 3 weeks after the prosthesis is placed in the body. This occurs 3 months before the high incidence of capsule contracture is observed, and it is more common within 1 year after surgery. In this study, we chose 45 days as the observation time point. With the addition of asiaticoside, TGF-β1 levels continued to decrease and were lowest in the carbon ion-implanted silicone rubber group. This indicates the advantages of carbon ion-implantation of silicone rubber combined with asiaticoside for reducing the incidence of capsular contracture.

Although the exact pathogenesis of capsular contracture needs to be further studied, the inflammatory response appears to play a role. Therefore, some scholars believe that changing the inflammatory response through drug treatment can reduce the occurrence of capsular contracture. Animal studies revealed that when 5 mg/kg leukotriene antagonist zarlukast is injected daily around the rough silicone rubber material, the capsule around the silicone rubber is thinner, the vascularization of the capsule is more abundant, and the collagen density is lower, indicating that this drug can prevent capsule contracture (Hinz et al., 2001; Mazzocchi et al., 2012). A study involving 60 patients with mild and severe capsular contracture evaluated monthly up to a year after the initiation of a 6-months treatment with zarlukast revealed that during active treatment, the Baker score of the contracture decreased, after the patient stopped treatment, the score began to increase (Bastos et al., 2012). This result suggests that to obtain long-term effects, long-term treatment is necessary. However, such long-term treatment with zarlukast can cause adverse reactions, such as adverse effects on liver function. In this study, the use of asiaticoside not only reduced collagen deposition but also reduced the thickness of the capsule and the probability of capsule contracture. The required treatment time with asiaticoside is also shorter, and the toxicity and side effects of the asiaticoside are reduced.

In conclusion, surface modification of silicone rubber materials with carbon ion implantation and combined application of asiaticoside regulates the proliferation and migration of fibroblasts through the TGF-β/Smad signaling pathway and reduces capsule thickness and collagen deposition. The application of these therapeutic methods could greatly reduce the incidence of capsule contracture. Based on these findings, the combined application of carbon silicone rubber and asiaticoside has potential in soft tissue filling procedures.

## Data Availability

The original contributions presented in the study are included in the article/Supplementary Material, further inquiries can be directed to the corresponding authors.
